# The complete mitochondrial genome of the hydrozoan jellyfish *Turritopsis lata* Lendenfeld, 1885 (Cnidaria; Hydrozoa; Anthoathecata) with molecular phylogenetic analysis

**DOI:** 10.1080/23802359.2021.1938725

**Published:** 2021-06-14

**Authors:** Yoseph Seo, Jinho Chae, Jang-Seu Ki

**Affiliations:** a Department of Biotechnology, Sangmyung University, Seoul, South Korea; b Marine Environmental Research and Information Laboratory, Gunpo, South Korea

**Keywords:** Hydrozoa, *Turritopsis lata*, mitochondrial genome, molecular phylogeny tree

## Abstract

In this study, we sequenced and analyzed the complete mitochondrial genome (mtgenome) of the hydrozoan jellyfish *Turritopsis lata*. The mtgenome was a complete linear form (15,047 bp in length, 30.9% A, 42.1% T, 12.5% C, and 14.5% G), including 13 protein coding genes (PCGs) (*cox*1*, cox*2*, cox*3*, atp*6*, atp*8*, nad*1*, nad*2*, nad*3*, nad*4*, nad*4L*, nad*5*, nad*6, and *cyt*b), 2 tRNAs (tRNA^Met^ and tRNA^Trp^), and 2 rRNAs (12S and 16S rRNA). The genome structure of the *T. lata* was completely identical to those of other species within the subclass Hydroidolina. In addition, our molecular phylogenetic analysis using 13 PCGs within hydrozoans showed that *T. lata* was the closest to *Turritopsis dohrnii*.

The hydrozoan jellyfish *Turritopsis* are well-known to revert their life cycle to the juvenile polyp stage from adult medusae by rejuvaniation, and, thus, they are called ‘immortal jellyfish’ (Hasegawa et al. 2016). The jellyfishes are recorded worldwide from tropical to temperate waters (Miglietta et al. 2007), and their distribution patterns are region-specific depending on species (Miglietta et al. 2019). Although they are classified by morphology, their identification is difficult due to similar morphology among relatives (Kubota 2015). Alternatively, molecular analysis has been considered as a powerful tool to determine their taxonomic identities (Li et al. 2018), and mitochondrial genes and genomes are commonly used as molecular markers of taxonomy (Yuan et al. 2016; Hashemi-Aghdam et al. 2017). To date, six *Turritopsis* species have been recorded in the public database (WoRMS 2021); of them, a mtgenome sequence was revealed only in *Turritopsis dohrnii* (KT020766). Additional molecular data are required for more accurate phylogenetic analysis of their taxa (Miglietta et al. 2007). In the present study, we report the mtgenome sequence and structure of *Turritopsis lata* Lendenfeld, 1885 (Cnidaria; Hydrozoa) with description of phylogenetic relationships within hydrozoans.

The *Turritopsis lata* specimen was collected from Tando Bay (34°58′56.4"N, 126°19′47.6"E), South Korea, on 1 September 2015. Total genomic DNA (gDNA) was extracted from the whole tissue using the modified cetyl-trimethylammonium bromide (CTAB) method (Richards et al. 2003). The remaining parts of the specimen and gDNA were stored in the specimen room of Department of Biotechnology (Dr. Hansol Kim, 201934001@sangmyung.kr), Sangmyung University, Korea, under the voucher number RH32. The complete mtgenome was sequenced on MGISEQ-200 platforms, and paired end reads of mtgenome sequences were assembled and annotated using GetOrganelle v1.7.1a (Jin et al. 2020), Geneious 9.1.3 (Geneious, Auckland, New Zealand), and MITOS (Bernt et al. 2013), respectively. A maximum-likelihood (ML) tree (JTT matrix-based model; 1000 bootstrap replication) was generated based on concatenated amino acid sequences of 13 PCGs in MEGA X (Kumar et al. 2018).

The complete mtgenome of *T. lata* (GenBank accession no. MW399220) was linear in shape and 15,047 bp in length with 73% AT content. The genome contained 13 PCGs (*cox*1*, cox*2*, cox*3*, atp*6*, atp*8*, nad*1*, nad*2*, nad*3*, nad*4*, nad*4L*, nad*5*, nad*6, and *cyt*b), two rRNAs (12S and 16S rRNA), and two tRNAs (tRNA^Met^ and tRNA^Trp^). The arrangement of 17 mitochondrial genes of *T. lata* was completely identical to another order Anthoathecata species, including *T. dohrnii* (KT020766), *Clava multicornis* (JN700935) and *Hydra oligactis* (EU237491) (Seo et al. 2020). Mitochondrial PCGs of *T. lata* have two start codons (ATG/GTG) and three stop codons (TAA/TAG/incomplete T). Especially, the incomplete T stop codon was found only in *cox*1.

The phylogenetic relationship within hydrozoans was inferred using amino acid sequences of 13 PCGs (Figure 1). The ML tree showed that the *T. lata* formed a sister relationship with *T. dohrnii*. In the present study, we provide additional complete mtgenome sequence data of *T. lata* to understand the abstruse phylogenetic relationship of hydrozoans.

**Figure 1.**
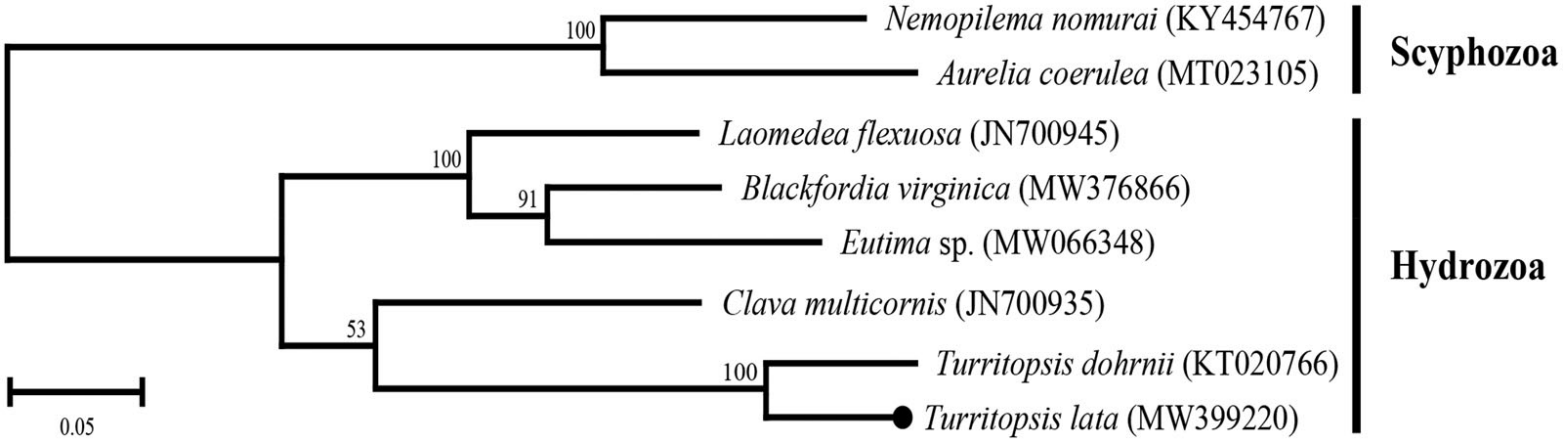
Molecular phylogenetic relationship of Hydrozoa. Two scyphozoans (*Nemopilema nomurai* and *Aurelia coerulea*) were included as the out-group. The maximum-likelihood (ML) phylogeny tree (JTT matrix-based model) was generated with the concatenated amino acid sequences of 13 mitochondrial PCGs. The percentage of replication value of bootstrap test (1000 replications) are shown above the branches. A black dot represents *Turritopsis lata* determined in this study.

## Data Availability

The genome sequence data that support the findings of this study are openly available in GenBank of NCBI at https://www.ncbi.nlm.nih.gov under the Accession no. MW399220.
